# The relation between oral health, depression, and anxiety among adults in Riyadh city, Saudi Arabia: a cross-sectional study

**DOI:** 10.3389/froh.2026.1850513

**Published:** 2026-06-12

**Authors:** Khalid A. Bin Abdulrahman, Abdulrahman R. Khouqeer, Abdullah M. Almousa, Fahad S. Alonazi, Abdulrahman A. Alsughayyir, Saud A. Alsahli

**Affiliations:** 1 Department of Medical Education, College of Medicine, Imam Mohammad Ibn Saud Islamic University (IMSIU), Riyadh, Saudi Arabia; 2 Department of Family Medicine, College of Medicine, Imam Mohammad Ibn Saud Islamic University (IMSIU), Riyadh, Saudi Arabia

**Keywords:** anxiety, cross-sectional study, depression, mental health, oral health-related quality of life, Saudi Arabia

## Abstract

**Background:**

Growing evidence suggests a bidirectional relationship between mental and oral health, with psychological distress having a detrimental impact on oral health behaviors and outcomes, and poor oral health exacerbating mental health symptoms. This study aimed to examine the relationship between depressive and anxiety symptoms and oral health-related quality of life (OHRQoL) among adults in Riyadh, Saudi Arabia.

**Methods:**

A cross-sectional study was conducted using a self-administered online questionnaire distributed to adults residing in Riyadh. The survey incorporated validated instruments: the Patient Health Questionnaire-9 (PHQ-9) to assess depressive symptoms, the Generalized Anxiety Disorder-7 (GAD-7) scale to measure anxiety symptoms, and the Oral Health Impact Profile-14 (OHIP-14) to evaluate OHRQoL. Descriptive statistics and multivariable linear regression analyses were carried out using RStudio (version 2024.9.1.394, Boston, MA, USA) with R version 4.4.2. Statistical significance was set at *p* < 0.05.

**Results:**

The analysis included 650 participants. Moderate-to-severe anxiety and depression were reported by 33.7% and 41.1% of participants, respectively. Higher PHQ-9 and GAD-7 scores were significantly associated with higher OHIP-14 scores (*p* < 0.001). In multivariable regression analysis, severe depression (*β* = 8.24) and severe anxiety (*β* = 5.71) demonstrated the strongest associations with increased OHIP-14 scores. Additionally, age ≥ 45 years and monthly family income between 10,000 and 20,000 SAR were independently associated with poorer OHRQoL.

**Conclusion:**

Severe depression and anxiety symptoms were independently associated with poorer OHRQoL among adults in Riyadh. These findings support integrating mental health assessments into oral healthcare services to enhance comprehensive, patient-centered care.

## Introduction

Mental health disorders represent a significant global public health challenge and rank among the leading contributors to years lived with disability worldwide. The World Health Organization estimates that more than one billion individuals experience mental health conditions, with depression and anxiety disorders among the most prevalent and debilitating (1). In addition to their psychological manifestations, these conditions are increasingly recognized as systemic disorders associated with impaired physical health, diminished functional capacity, and reduced overall quality of life (1, 2). National survey data in Saudi Arabia indicate that approximately one-third of the population will experience a mental health disorder at some point in their lives, with substantially higher prevalence observed among women (3). These findings underscore the epidemiological importance of mental health within the region.

**Figure 1:**
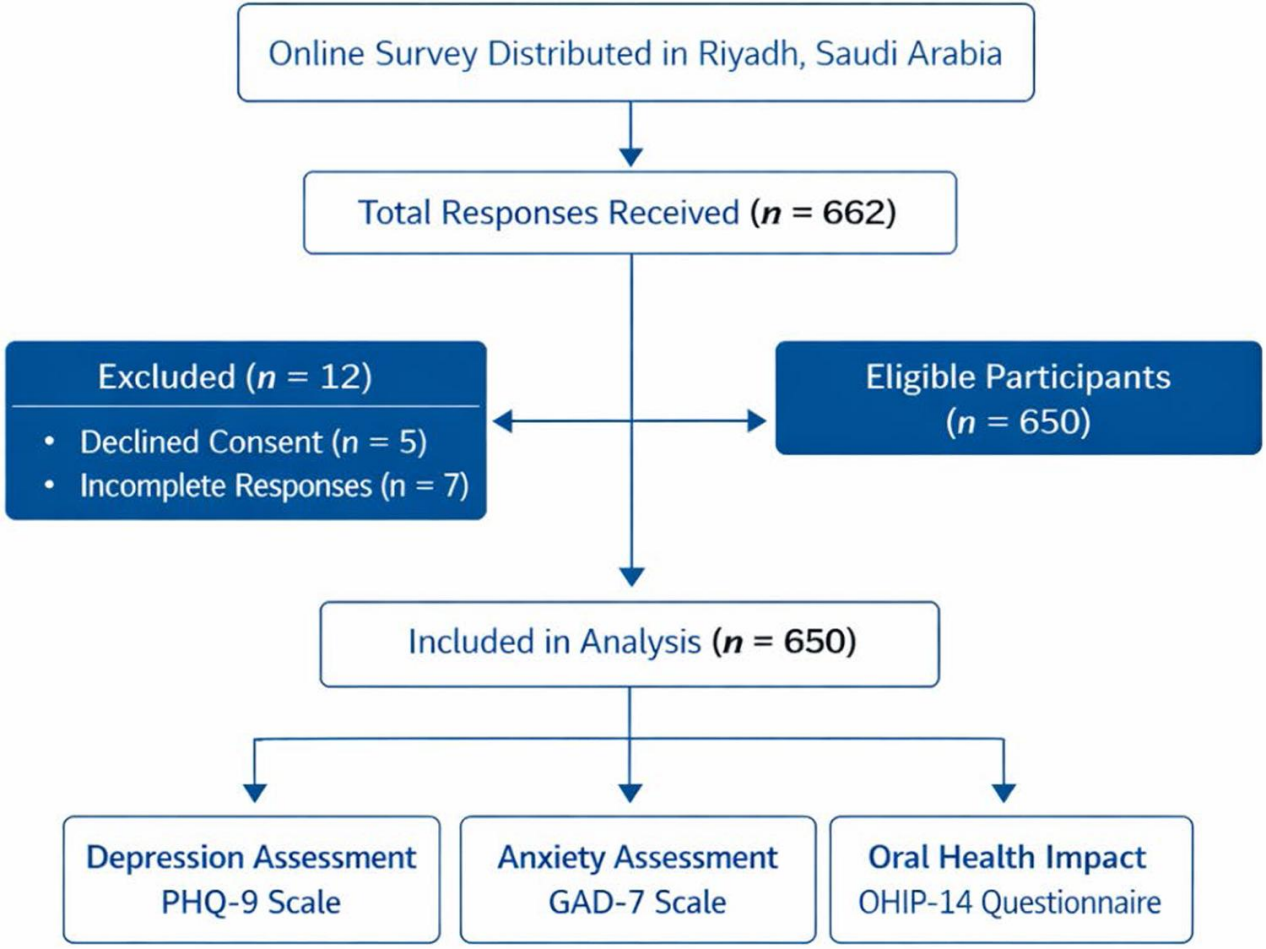
Flow diagram illustrating the recruitment and selection process of participants in this cross-sectional study conducted in Riyadh, Saudi Arabia (October–November 2025). A total of 662 responses were received through an online survey. Twelve responses were excluded due to incomplete questionnaires (*n* = 7) or declined consent (*n* = 5), resulting in 650 eligible participants included in the final analysis. All included participants completed validated assessments of depressive symptoms (PHQ-9), anxiety symptoms (GAD-7), and oral health-related quality of life (OHIP-14).

Concurrently, oral diseases remain highly prevalent globally and are recognized as a significant public health concern. Oral health extends beyond the mere absence of dental pathology and encompasses functional, psychosocial, and social well-being (4, 5). The concept of oral health-related quality of life (OHRQoL) has therefore emerged as a multidimensional measure capturing the impact of oral conditions on daily activities, emotional status, and social participation (6, 7). OHRQoL has become a central outcome measure in oral epidemiology, reflecting patient-centered perspectives and enabling a more comprehensive evaluation of the burden of oral disease (8).

A growing body of research suggests that mental and oral health are interrelated through complex behavioral, biological, and social mechanisms. Depression and anxiety may adversely affect oral health through behavioral pathways such as reduced oral hygiene practices, irregular dental attendance, and unhealthy dietary patterns (9). Physiological mechanisms, including altered salivary flow, inflammatory responses, and stress-related parafunctional habits, may also contribute to the progression of oral disease. Conversely, oral pain, functional impairment, and aesthetic concerns may exacerbate psychological distress, suggesting a potentially bidirectional relationship (8, 9).

Andersen's behavioral model of health services use provides a useful theoretical framework for understanding these interrelationships (10). The model posits that health outcomes are shaped by the interaction of predisposing characteristics, enabling resources, and perceived health needs. Depression may function both as a predisposing and need-related factor that influences health behaviors and healthcare utilization, thereby affecting oral health outcomes and OHRQoL. At the same time, poor oral health may intensify perceived need and psychological burden, reinforcing a reciprocal cycle. Integrating this theoretical framework strengthens the epidemiological rationale for examining mental health as a determinant of OHRQoL in population-based studies (10).

Despite increasing international attention to this relationship, several methodological limitations remain. For example, many studies rely on general psychological distress instruments that do not distinguish between depressive and anxiety symptoms, limiting causal interpretation and specificity (8, 11). Furthermore, research conducted in Saudi Arabia has largely focused on university students or patients with specific chronic conditions (9, 12, 13). Although these studies provide important preliminary insights, their findings may not be generalizable to the broader adult population. As a result, studies employing validated disorder-specific instruments in broader adult samples remain limited. Given the high prevalence of both mental health disorders and oral health problems in Saudi Arabia, understanding their intersection has important implications for integrated public health strategies and patient-centered care. Disentangling the independent contributions of depressive and anxiety symptoms is particularly important, as these conditions frequently co-occur but may exert differential effects on health behaviors and quality of life (11).

Accordingly, the present study aimed to examine the association between depressive and anxiety symptoms and OHRQoL among adults in Riyadh, Saudi Arabia. By employing validated disorder-specific measures—the PHQ-9, GAD-7, and OHIP-14 scales.

## Methods

### Study design and setting

A cross-sectional study was conducted to examine the association between mental health symptoms and OHRQoL among adults residing in Riyadh, Saudi Arabia. Data were collected through a self-administered online questionnaire distributed from October 16 to November 6, 2025, using Microsoft Forms. The survey link was disseminated through institutional and social media networks without paid promotion, targeting, or advertisement boosting. The questionnaire was administered in Arabic using validated Arabic versions of all measurement instruments. Participation was voluntary, and electronic informed consent was obtained from all respondents before they completed the survey.

The study protocol was reviewed and approved by the Institutional Review Board (IRB) of Imam Mohammad Ibn Saud Islamic University (Project No. 876-2025; approval date: September 11, 2025), and the study was conducted in accordance with the principles of the Declaration of Helsinki. The manuscript was prepared in accordance with the Strengthening the Reporting of Observational Studies in Epidemiology (STROBE) reporting guidelines for cross-sectional studies.

### Participants and eligibility criteria

Participants were recruited from the general adult population of Riyadh Province, Saudi Arabia. Eligible participants were individuals aged 18 years or older residing in Riyadh at the time of data collection and were able to access and complete the online survey.

Individuals residing outside Riyadh, those younger than 18 years, and respondents who declined to provide consent were excluded from the study. Incomplete questionnaires were also excluded from the final analysis.

### Sample size calculation

The minimum required sample size was calculated using the Raosoft online sample size calculator. Assuming a population size of approximately 8 million residents in Riyadh Province, a 95% confidence level, and a 5% margin of error, the estimated minimum required sample size was 385 participants. A total of 662 individuals responded to the survey. After excluding 12 respondents who either did not complete the questionnaire or declined to participate, 650 participants were included in the final analysis, which exceeded the minimum required sample size. Although the primary sample size estimation was based on prevalence assumptions, the final sample size of 650 participants was also considered adequate for the planned multivariable regression analyses, providing sufficient observations per predictor category to minimize model overfitting and ensure stable parameter estimation. The participant recruitment and selection process is illustrated in Figure 1.

### Data collection and study instruments

The structured questionnaire consisted of five sections:

- Informed consent: An introductory section describing the study objectives, voluntary participation, and assurances of confidentiality.
- Sociodemographic characteristics: Age, sex, educational level, occupation, nationality, and monthly family income.
- Oral health-related quality of life: Assessed using the OHIP-14 scale, a validated instrument measuring the functional, psychological, and social impacts of oral conditions.
- Depressive symptoms: Evaluated using the PHQ-9 scale, a widely used and validated measure of depression severity.
- Anxiety symptoms: Assessed using the GAD-7 scale, a validated instrument measuring anxiety severity.

All instruments have established psychometric validity and reliability and were administered using validated Arabic versions of the PHQ-9, GAD-7, and OHIP-14 instruments previously validated in Arabic-speaking populations were used (12–14). PHQ-9 scores were categorized into minimal, mild, moderate, moderately severe, and severe depressive symptom groups according to established cut-off values (0–4, 5–9, 10–14, 15–19, and 20–27, respectively). GAD-7 scores were categorized into minimal, mild, moderate, and severe anxiety levels using standard thresholds (0–4, 5–9, 10–14, and 15–21, respectively). To minimize duplicate responses, the survey platform restricted multiple submissions from the same electronic device.

### Statistical analysis

Given the cross-sectional design and convenience-based online recruitment approach, all analyses were conducted using a complete-case framework without applying sampling weights. As probability-based sampling was not employed, inferential analyses were interpreted as reflecting associations within the study sample rather than population-level prevalence estimates. Multivariable linear regression was retained because residual diagnostics indicated acceptable fit to the model assumptions despite the non-normal distribution of raw OHIP-14 scores, and because linear regression models are generally robust to moderate deviations from normality in large samples. Standard regression diagnostics were performed to assess model assumptions, including linearity, homoscedasticity, and absence of multicollinearity.

All statistical analyses were performed using RStudio (version 2024.9.1.394, Boston, MA, USA) and R (version 4.4.2). Descriptive statistics were used to summarize participant characteristics and study variables. Categorical variables were presented as frequencies and percentages. Continuous variables were evaluated for distributional characteristics and are reported as medians with interquartile ranges (IQR), given the non-normal distribution of OHIP-14 scores. The internal consistency reliability of the PHQ-9, GAD-7, and OHIP-14 scales was evaluated using Cronbach's alpha coefficients.

Comparisons of OHIP-14 scores across sociodemographic categories and mental health severity groups were conducted using nonparametric tests. The Wilcoxon rank-sum test was applied for two-group comparisons, and the Kruskal–Wallis test was used for comparisons involving more than two groups.

Multivariable linear regression was retained because residual diagnostics demonstrated acceptable approximation of model assumptions despite the non-normal distribution of raw OHIP-14 scores. Furthermore, linear regression models are generally robust to moderate deviations from normality in sufficiently large samples. Variables with clinical or statistical relevance were included in the regression model. Regression coefficients (β) with 95% confidence intervals (CIs) were reported. Variance inflation factor (VIF) values were examined for all regression predictors; all were below 2.5, indicating no evidence of problematic multicollinearity between PHQ-9 and GAD-7 variables.

### Subgroup and sensitivity analyses

Pre-specified subgroup analyses were conducted to examine the consistency of associations between mental health severity and OHRQoL across age groups and gender categories. In addition, sensitivity analyses were performed by modeling PHQ-9 and GAD-7 scores as continuous variables rather than categorical severity groups to assess the robustness of the observed associations. All statistical tests were two-sided, and *p*-values < 0.05 were considered statistically significant.

## Results

### Participant characteristics

A total of 662 responses were received. After excluding 12 incomplete or non-consented questionnaires, 650 participants were included in the final analysis.

The sample comprised 332 males (51.1%) and 318 females (48.9%). The majority of participants were aged 18–24 years (66.2%), followed by those aged ≥ 45 years (13.7%). Over 50.2% held a bachelor's degree, while 35.8% had educational attainment below high school level. Most participants were students (61.7%), and 24.3% were employed. Nearly all respondents were Saudi nationals (96.9%). Regarding monthly family income, 27.4% reported earning less than 5,000 SAR, while 28.6% preferred not to disclose their income (Table 1).

**Table 1:** Demographic characteristics of the participants.

Characteristic	Description
Gender	
Male	332 (51.1%)
Female	318 (48.9%)
Age	
18–24	430 (66.2%)
25–34	74 (11.4%)
35–44	57 (8.8%)
45 or more	89 (13.7%)
Highest educational level	
Below high school	233 (35.8%)
High school diploma	46 (7.1%)
Bachelor	326 (50.2%)
Master	29 (4.5%)
PhD	16 (2.5%)
Occupation	
Student	401 (61.7%)
Unemployed	28 (4.3%)
Employed	158 (24.3%)
Housewife	34 (5.2%)
Retired	29 (4.5%)
Nationality	
Saudi	630 (96.9%)
Non-Saudi	20 (3.1%)
Family income (SAR)	
< 5,000	178 (27.4%)
5,000 to 10,000	82 (12.6%)
10,000 to 20,000	114 (17.5%)
> 20,000	90 (13.8%)
Prefer not to say	186 (28.6%)

*n* (%)

**Table 2:** Description of the domains and subdomains in the current study.

Characteristic	Median (Q1 - Q3)	Mean ± SD	Min - Max	Cronbach Alpha	N of items
Anxiety (GAD-7)	7.0 (4.0–12.0)	8.0 ± 5.8	0.0–21.0	0.918	7
Depression (PHQ-9)	9.0 (4.0–14.0)	9.4 ± 6.8	0.0–27.0	0.905	9
Quality of life related to oral health (OHIP-14)	11.0 (5.0–19.0)	12.8 ± 10.0	0.0–51.0	0.914	14

### Distribution and reliability of mental health and OHRQoL measures

The median scores were 7.0 (IQR 4.0–12.0) for GAD-7, 9.0 (IQR 4.0–14.0) for PHQ-9, and 11.0 (IQR 5.0–19.0) for OHIP-14. All instruments demonstrated excellent internal consistency, with Cronbach's alpha values of 0.918 (GAD-7), 0.905 (PHQ-9), and 0.914 (OHIP-14). Descriptive statistics and internal consistency reliability coefficients for the study instruments are presented in Table 2.

### Severity categories of anxiety and depression

Based on established cut-off scores, mild anxiety was the most prevalent category (36.5%), followed by minimal anxiety (29.8%), moderate anxiety (18.2%), and severe anxiety (15.5%). For depressive symptoms, mild depression was the most common (32.0%), followed by minimal depression (25.8%), moderate depression (19.4%), moderately severe depression (13.8%), and severe depression (8.9%). Overall, moderate-to-severe levels of anxiety and depression were observed in 33.7% and 41.1% of participants, respectively.

### Item-level distribution of PHQ-9 and GAD-7 responses

At the item level, the GAD-7 symptoms most frequently reported as occurring “nearly every day” were excessive worrying (20.5%) and feeling nervous, anxious, or on edge (17.2%). For the PHQ-9, the most commonly reported symptoms at the same frequency were fatigue or low energy (20.5%) and sleep disturbances, including difficulty falling or staying asleep (20.2%).

**Figure 2:**
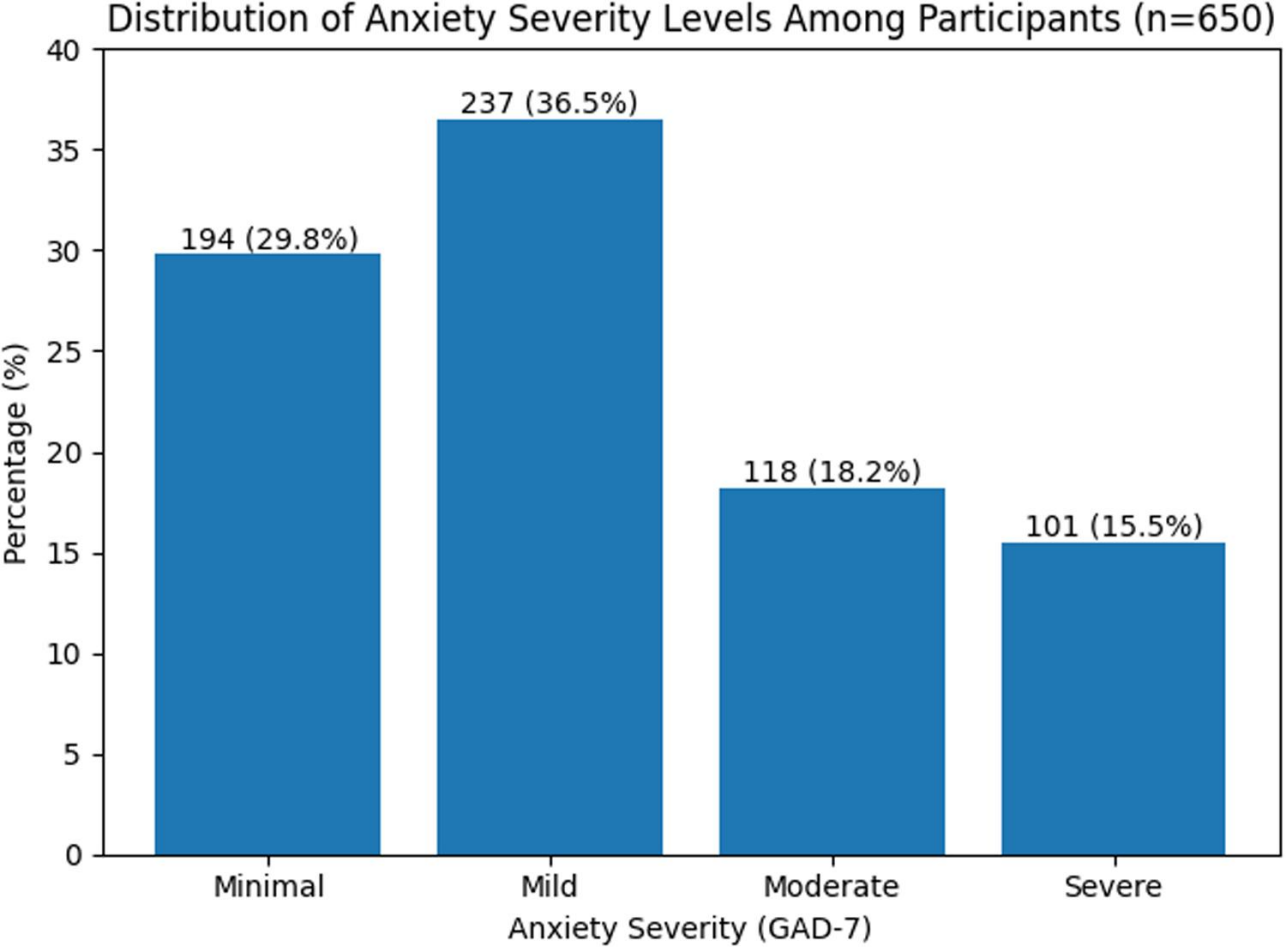
Distribution of anxiety severity levels among participants based on GAD-7 scores.

Figure 2 illustrates the distribution of anxiety severity levels among the 650 study participants, categorized according to the GAD-7 scoring criteria. Mild anxiety was the most frequently reported category (*n* = 237, 36.5%), followed by minimal anxiety (*n* = 194, 29.8%). Moderate anxiety was reported by 118 participants (18.2%), while severe anxiety was observed in 101 participants (15.5%). Overall, 33.7% of participants exhibited moderate-to-severe anxiety symptoms.

**Figure 3:**
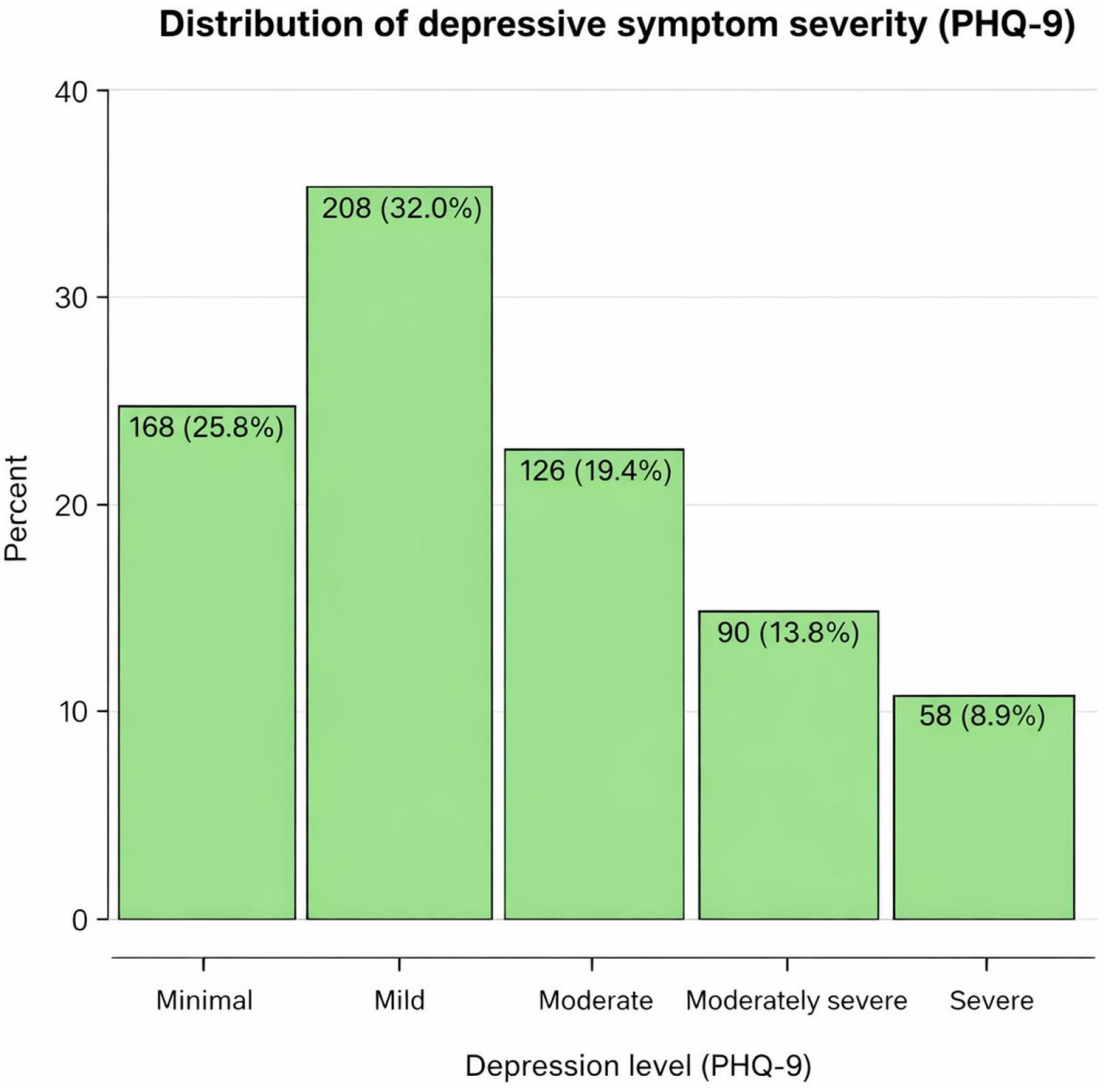
Distribution of depressive symptom severity among participants based on PHQ-9 scores.

Figure 3 presents the distribution of depressive symptom severity among the 650 study participants, stratified according to PHQ-9 classification. Mild depression was the most frequently reported category (*n* = 208, 32.0%), followed by minimal depression (*n* = 168, 25.8%). Moderate depression was reported by 126 participants (19.4%), moderately severe depression by 90 participants (13.8%), and severe depression by 58 participants (8.9%). Overall, 41.1% of participants exhibited moderate-to-severe depressive symptoms. Depressive symptom categories were classified according to standard PHQ-9 severity thresholds, including minimal symptom scores, which do not necessarily indicate clinically significant depression.

### OHIP-14 domain responses

Across the OHIP-14 domains, the impacts most frequently reported as occurring “most of the time” or “always” were related to physical pain (painful aching and discomfort while eating), psychological disability (embarrassment and difficulty relaxing), and psychological discomfort (feeling tense). Smaller proportions of participants reported functional limitations (difficulty pronouncing words or altered taste), social disability (irritability), and handicap (reduced life satisfaction or inability to function).

Detailed item-level distributions are presented in Table 3.

**Table 3:** Participants’ responses to the OHIP questionnaire.

Characteristic	Never	Rarely	Sometimes	Most of the time	Always
Functional limitation					
Pronouncing words	377 (58.0%)	119 (18.3%)	119 (18.3%)	25 (3.8%)	10 (1.5%)
Sense of taste worsened	381 (58.6%)	142 (21.8%)	109 (16.8%)	14 (2.2%)	4 (0.6%)
Physical pain					
Painful aching (mouth)	207 (31.8%)	180 (27.7%)	197 (30.3%)	55 (8.5%)	11 (1.7%)
Uncomfortable eating any foods	171 (26.3%)	155 (23.8%)	235 (36.2%)	58 (8.9%)	31 (4.8%)
Psychological discomfort					
Been self-conscious	419 (64.5%)	135 (20.8%)	74 (11.4%)	16 (2.5%)	6 (0.9%)
Felt tense	269 (41.4%)	133 (20.5%)	158 (24.3%)	48 (7.4%)	42 (6.5%)
Physical disability					
Diet been unsatisfactory	450 (69.2%)	79 (12.2%)	91 (14.0%)	13 (2.0%)	17 (2.6%)
Interrupt meals	255 (39.2%)	175 (26.9%)	166 (25.5%)	42 (6.5%)	12 (1.8%)
Psychological disability					
Difficult to relax	224 (34.5%)	186 (28.6%)	179 (27.5%)	36 (5.5%)	25 (3.8%)
Feel a bit embarrassed	267 (41.1%)	123 (18.9%)	166 (25.5%)	57 (8.8%)	37 (5.7%)
Social disability					
Irritable with people	304 (46.8%)	143 (22.0%)	122 (18.8%)	51 (7.8%)	30 (4.6%)
Difficulty doing the usual	358 (55.1%)	163 (25.1%)	106 (16.3%)	17 (2.6%)	6 (0.9%)
Handicap					
Life in general is less satisfying	333 (51.2%)	148 (22.8%)	125 (19.2%)	24 (3.7%)	20 (3.1%)
Totally unable to function	385 (59.2%)	165 (25.4%)	76 (11.7%)	15 (2.3%)	9 (1.4%)

### Predictors of OHRQoL

#### Bivariate analysis

Higher OHIP-14 scores were significantly associated with age group (*p* = 0.029), family income (*p* < 0.001), anxiety severity (*p* < 0.001), and depression severity (*p* < 0.001).

Participants aged ≥ 45 years had higher median OHIP-14 scores than those aged 18–24 years (14.0 vs. 10.0). Participants with a monthly family income between 10,000 and 20,000 SAR also had higher OHIP-14 scores than those with a monthly family income less than 5,000 SAR.

#### Multivariable regression analysis

In the adjusted multivariable model, age ≥ 45 years and monthly family income of 10,000–20,000 SAR were independently associated with higher OHIP-14 scores. Anxiety severity demonstrated a graded positive association with OHRQoL impairment, with moderate and severe anxiety showing particularly strong effects. Similarly, increasing depressive symptom severity was independently associated with higher OHIP-14 scores, with severe depression exhibiting the largest effect size. Full regression results are presented in Table 4. Correlation analyses between PHQ-9 and GAD-7 scores demonstrated moderate positive correlation without evidence of excessive collinearity, supporting their simultaneous inclusion in the multivariable model.

**Table 4:** Factors and predictors of higher OHIP scores (poor quality of life related to oral health).

Variable	OHIP score		Multivariable regression		
	Median (IQR)	*p*-value	Beta	95% CI	*p*-value
Gender		0.003			
Male	10.0 (4.0, 18.0)		Reference	Reference	
Female	12.0 (6.0, 20.0)		1.17	−0.40, 2.73	0.146
Age		0.029			
18 to 24	10.0 (4.0, 18.0)		Reference	Reference	
25 to 34	13.5 (7.0, 18.0)		1.23	−2.10, 4.56	0.471
35 to 44	13.0 (6.0, 22.0)		2.34	−1.59, 6.26	0.244
45 or more	14.0 (6.0, 22.0)		3.92	0.07, 7.77	0.047
Highest educational level		0.382			
Below high school	10.0 (4.0, 19.0)		Reference	Reference	
High school diploma	14.5 (4.0, 25.0)		1.42	−1.63, 4.48	0.361
Bachelor	11.0 (6.0, 18.0)		0.06	−1.61, 1.73	0.940
Master	13.0 (6.0, 21.0)		−0.54	−4.53, 3.45	0.791
PhD	12.5 (4.5, 22.0)		1.60	−3.45, 6.65	0.536
Occupation		0.250			
Student	10.0 (4.0, 18.0)		Reference	Reference	
Unemployed	13.5 (5.0, 22.5)		0.72	−3.26, 4.70	0.723
Employed	13.0 (6.0, 20.0)		0.53	−2.81, 3.86	0.758
Housewife	10.5 (6.0, 24.0)		1.17	−3.29, 5.63	0.608
Retired	15.0 (5.0, 22.0)		1.53	−3.71, 6.77	0.568
Nationality		0.133			
Saudi	11.0 (5.0, 19.0)		Reference	Reference	
Non-Saudi	14.5 (10.0, 21.5)		2.21	−1.94, 6.36	0.298
Family income (SAR)		< 0.001			
< 5,000	10.0 (4.0, 19.0)		Reference	Reference	
5,000 to 10,000	14.5 (6.0, 24.0)		2.20	−0.37, 4.77	0.094
10,000 to 20,000	15.0 (7.0, 22.0)		2.42	0.07, 4.78	0.044
> 20,000	7.0 (3.0, 17.0)		−1.19	−3.61, 1.23	0.337
Prefer not to say	11.0 (5.0, 18.0)		−0.22	−2.13, 1.69	0.822
Anxiety level		< 0.001			
Minimal	7.0 (2.0, 13.0)		Reference	Reference	
Mild	12.0 (5.0, 18.0)		2.99	1.00, 4.97	0.003
Moderate	15.0 (8.0, 24.0)		6.48	3.82, 9.13	< 0.001
Severe	17.0 (8.0, 26.0)		5.71	2.51, 8.90	< 0.001
Depression level		< 0.001			
Minimal	6.0 (1.5, 13.0)		Reference	Reference	
Mild	12.0 (5.0, 19.0)		3.34	1.30, 5.38	0.001
Moderate	12.0 (6.0, 20.0)		3.09	0.58, 5.61	0.016
Moderately severe	15.5 (7.0, 25.0)		4.42	1.46, 7.38	0.004
Severe	18.5 (10.0, 30.0)		8.24	4.54, 12.0	< 0.001

IQR, interquartile range.

Wilcoxon rank sum test; Kruskal–Wallis rank sum test.

CI, Confidence Interval.

#### Subgroup and sensitivity analyses

The positive association between depressive and anxiety symptom severity and OHIP-14 scores remained consistent across gender and age strata, with no statistically significant interaction effects observed. Sensitivity analyses in which PHQ-9 and GAD-7 scores were treated as continuous variables yielded associations of similar direction and magnitude, confirming the robustness of the primary findings.

## Discussion

### Principal findings

This study aimed to examine the association between OHRQoL, depression, and anxiety among adults residing in Riyadh using validated disorder-specific instruments (PHQ-9 and GAD-7), alongside the OHIP-14. The findings provide a detailed assessment of the relationship between mental health symptoms and self-reported oral health-related quality of life in the Saudi context, addressing limitations identified in prior regional research. Moderate-to-severe anxiety and depression were observed in 33.7% and 41.1% of participants, respectively. The median GAD-7 score was 7.0, indicating mild anxiety, and the median PHQ-9 score was 9.0, corresponding to mild depressive symptoms.

A robust and graded association was identified between increasing severity of anxiety and depression symptoms and poorer OHRQoL, reflected by higher OHIP-14 scores. Multivariable regression analysis confirmed that greater levels of anxiety and depression were independently associated with reduced OHRQoL. Severe depression demonstrated the strongest effect size, and frequently reported symptoms included excessive worry and nervousness for anxiety and fatigue and sleep disturbances for depression. Within the OHIP-14 domains, embarrassment and discomfort while eating were among the most commonly reported impacts. Sociodemographic factors, including age ≥ 45 years and family income between 10,000 and 20,000 SAR, were independently associated with higher OHIP-14 scores, warranting further contextual investigation. These findings are consistent with previous research conducted among university students in Riyadh, which demonstrated significantly higher OHIP-14 scores among individuals reporting severe depression and anxiety (12). These findings should be interpreted within the context of the study sample, which was predominantly composed of younger adults and students recruited through convenience-based online sampling.

### Interpretation within the epidemiological and theoretical context

The prevalence of anxiety and depressive symptoms observed in this study is consistent with global and national trends, indicating a substantial burden of mental health conditions (14). Although median scores reflected mild symptom levels, a considerable proportion of participants reported moderate-to-severe symptoms, indicating clinically meaningful psychological distress.

The significant independent association between anxiety, depression, and poorer OHRQoL may be explained through behavioral, physiological, and psychosocial mechanisms. Individuals experiencing psychological distress may neglect oral hygiene practices, reduce dental attendance, and adopt unhealthy dietary behaviors (15). Fatigue and sleep disturbance may further compromise self-care behaviors. Physiological mechanisms proposed in prior literature suggest that psychological distress may alter salivary function, contribute to xerostomia, and increase stress-related bruxism; however, these mechanisms were not directly assessed in the present study (16). The prominence of painful oral sensations and eating discomfort in the OHIP-14 responses supports the contribution of tangible somatic manifestations. In addition, the frequent reporting of embarrassment suggests that psychosocial consequences of oral conditions may reinforce a negative feedback cycle between mental distress and perceived oral health impact.

These findings align with Andersen's behavioral model, in which depression and anxiety may act as predisposing and need-related factors influencing health behaviors and healthcare utilization (10, 11). The reciprocal relationship between psychological distress and oral health impairment supports a multidimensional conceptualization of OHRQoL. Given the cross-sectional nature of the study, the observed associations should not be interpreted as evidence of causality. Reverse causation and reciprocal influences between mental health symptoms and perceived oral health impact remain plausible.

### Comparison with existing literature

The present findings corroborate international research demonstrating strong associations between mental health disorders and poorer OHRQoL (17, 18). Systematic analyses have consistently reported that individuals with depression and anxiety disorders experience significantly diminished OHRQoL compared with the general population (16).

Within the Saudi context, previous studies have been constrained by either sampling limitations or reliance on general psychological distress instruments (8, 13). Studies conducted among university students in Riyadh have demonstrated associations between mental health status and subjective oral health outcomes (19). The current study extends these findings by examining a broader adult sample with validated, disorder-specific instruments (PHQ-9 and GAD-7), thus improving methodological rigor and comparability with international studies.

This non-linear association may reflect sample composition, the predominance of younger participants and students, or residual confounding from unmeasured socioeconomic and behavioral factors rather than a direct socioeconomic gradient.

### Clinical and public health implications

The findings have important implications for integrated healthcare delivery. The graded association between mental health symptom severity and OHRQoL supports the development of collaborative care models wherein dental and mental health services operate synergistically (20, 21). Brief screening tools such as PHQ-2 and GAD-2 may facilitate early identification of psychological distress within dental settings.

Oral health education programs tailored to individuals with depression and anxiety may help mitigate behavioral risk factors, particularly reduced motivation and fatigue affecting oral hygiene practices (22–25). Given the proportion of participants reporting moderate-to-severe symptoms, targeted public health initiatives are warranted to prevent deterioration of oral health and OHRQoL among high-risk groups (26, 27). Management strategies should also address somatic manifestations associated with psychological distress, such as bruxism and xerostomia, through preventive and supportive interventions (28–30).

### Limitations and future directions

Several limitations should be acknowledged. First, the cross-sectional design precludes causal inference, suggesting a possible bidirectional relationship between psychological distress and OHRQoL. Second, convenience sampling and online data collection may have introduced selection bias, leading to an overrepresentation of younger, more educated participants. Consequently, the findings should not be interpreted as population-representative estimates for the broader adult population of Riyadh or Saudi Arabia. Third, reliance on self-reported measures (PHQ-9, GAD-7, and OHIP-14) may have introduced recall or social desirability bias. Fourth, no clinical oral examination was conducted; therefore, findings reflect perceived oral health impact rather than objective clinical indices. Although subgroup and sensitivity analyses supported the robustness of the findings, residual confounding from unmeasured behavioral or clinical variables cannot be ruled out. Residual confounding from unmeasured variables, including smoking status, oral hygiene practices, dental attendance behaviors, systemic diseases, and medication use (particularly antidepressants associated with xerostomia), cannot be excluded.

Future longitudinal research is required to elucidate the temporal relationships between changes in mental health status and OHRQoL. Integrating objective clinical measures, such as the DMFT index and periodontal assessment, along with biological markers, including inflammatory markers and salivary parameters, would strengthen causal inference. Intervention studies evaluating integrated mental–oral healthcare approaches may further elucidate pathways for improving both psychological well-being and oral health outcomes.

## Conclusion

Among adults in Riyadh, greater severity of anxiety and depressive symptoms was independently associated with poorer OHRQoL. These findings underscore the public health significance of mental distress in shaping oral health outcomes and support the integration of mental and dental healthcare strategies within clinical and preventive frameworks.

## Data Availability

The raw data supporting the conclusions of this article will be made available by the authors, without undue reservation.

## References

[1] World Health Organization. World Mental Health Report: Transforming Mental Health for all. Geneva: World Health Organization (2022).

[2] Institute for Quality and Efficiency in Health Care (IQWiG). Depression: overview. InformedHealth.org (2020). Available online at: https://www.ncbi.nlm.nih.gov/books/NBK279285/ Accessed 30 November 2025. 23101074

[3] Saudi National Mental Health Survey. Saudi National Mental Health Survey: Technical Report. Riyadh: Ministry of Health (2019).

[4] Chen X Liu X Li F He H Li X Qin T. Depression and health outcomes: an umbrella review of systematic reviews and meta-analyses of observational studies. Transl Psychiatry. (2025) 15(1):298. 10.1038/s41398-025-03463-8 40835677 PMC12368087

[5] Machado MO Veronese N Sanches M Stubbs B Koyanagi A Thompson T. The association of depression and all-cause and cause-specific mortality: an umbrella review of systematic reviews and meta-analyses. BMC Med. (2018) 16(1):112. 10.1186/s12916-018-1101-z 30025524 PMC6053830

[6] Kisely S Baghaie H Lalloo R Siskind D Johnson NW. A systematic review and meta-analysis of the association between poor oral health and severe mental illness. Psychosom Med. (2015) 77(1):83–92. 10.1097/PSY.0000000000000135 25526527

[7] AlSuliman FS Zaazouee MS. Associations between mental health and oral health in Saudi Arabia: an online survey-based cross-sectional study. Cureus. (2022) 14:e31732. 10.7759/cureus.31732 36569720 PMC9769782

[8] Asiri A Nazir MA Alsharief M Shahin S Al-Ansari A KS A-K. Effect of psychological distress on oral health: a cross-sectional study. BMC Oral Health. (2024) 24(1):1508. 10.1186/s12903-024-05319-x 39702206 PMC11660662

[9] Alutaibi A Bakry S Albagami S Hadi M Aboalreesh A Fudah A. The association between oral health and depression among university students in Makkah city: a web-based survey study. Med Sci. (2023) 27(139):1–9. 10.54905/disssi.v27i139.e352ms3221

[10] Andersen RM. Revisiting the behavioral model and access to medical care: does it matter? J Health Soc Behav. (1995) 36(1):1. 10.2307/2137284 7738325

[11] Tiwari T Kelly A Randall CL Tranby E Franstve-Hawley J. Association between mental health and oral health status and care utilization. Front Oral Health. (2022) 2:732882. 10.3389/froh.2021.732882 35199101 PMC8859414

[12] AlJameel AH AlSaleh LS Bawazir NH AlOmair AS AlMalki SA. Association between oral health-related quality of life and mental health among university students in Riyadh, Saudi Arabia. Clin Epidemiol Glob Health. (2025) 33:101984. 10.1016/j.cegh.2025.101984

[13] Thirunavukkarasu A Alharbi MS Salahuddin M Al-Hazmi AH ALruwaili BF Alsaidan AA. Evaluation of oral health-related quality of life and its association with mental health status of patients with type 2 diabetes mellitus in the post-COVID-19 pandemic era: a study from central Saudi Arabia. Front Public Health. (2023) 11:1158979. 10.3389/fpubh.2023.1158979 37033065 PMC10080138

[14] World Health Organization. Mental health: strengthening our response (2025). Available online at: https://www.who.int/news-room/fact-sheets/detail/mental-health-strengthening-our-response Accessed 30 November 2025.

[15] Flavin K Paulson DR VanDeWiele M Evans M Stull C. Oral health status and oral health-related quality of life among a convenience sample of individuals receiving inpatient psychiatric care: a retrospective cross-sectional study. BMC Oral Health. (2025) 25(1):1135. 10.1186/s12903-025-06499-w 40634985 PMC12243241

[16] Hajek A. König H-H. Oral health-related quality of life, probable depression and probable anxiety: evidence from a representative survey in Germany. BMC Oral Health. (2022) 22(1):9. 10.1186/s12903-022-02047-y 35034663 PMC8761375

[17] Nerobkova N Park E-C. Jang S-I. Depression and oral health-related quality of life: a longitudinal study. Front Public Health. (2023) 11:1072115. 10.3389/fpubh.2023.1072115 36844860 PMC9947840

[18] Alimoradi Z Jafari E Roshandel Z Potenza MN Lin C-Y Pakpour AH. Meta-analysis with systematic review to synthesize associations between oral health related quality of life and anxiety and depression. BDJ Open. (2024) 10(1):9. 10.1038/s41405-024-00191-x 38350985 PMC10864408

[19] AlJameel A AlSaleh L Bawazir N AlOmair A Almalki S. How mental health correlates with subjective oral health status: a cross-sectional study among a group of university students. Niger J Clin Pract. (2023) 26(11):1716–22. 10.4103/njcp.njcp_330_23 38044778

[20] Khairunnisa Z Siluvai S Kanakavelan K Agnes L Kp I G K. Mental and oral health: a dual frontier in healthcare integration and prevention. Cureus. (2024) 16(12):e76264. 10.7759/cureus.76264 39845207 PMC11753583

[21] Heaton LJ Tiwari T Tranby EP. Medical-dental-behavioral integration: embracing whole-person health in research and practice. JDR Clin Transl Res. (2024) 9(1_suppl):3S. 10.1177/23800844241273799 39558732

[22] Berryhill MB Culmer N Smith TB McBurnie A Barton M Machen D. Patient perceptions on the acceptability and appropriateness of mental health screening and follow-up in national dental practice-based research network practices. Transl Behav Med. (2025) 15(1):ibaf022. 10.1093/tbm/ibaf022 40512532 PMC12169344

[23] Khokhar MA Khokhar WA Clifton AV Tosh GE. Oral health education (advice and training) for people with serious mental illness. Cochrane Database Syst Rev. (2016) 2016(9)9(9):CD008802. 10.1002/14651858.CD008802.pub3 PMC6457656 27606629

[24] CentraDental. The connection between mental health and oral health (2024). Available online at: https://www.centrasotadental.com/the-connection-between-mental-health-and-oral-health.php Accessed 30 November 2025.

[25] Pecka L. From mood to mouth: the surprising effects of mental health on teeth (2025). Available online at: https://progressivedentalny.com/from-mood-to-mouth-the-surprising-effects-of-mental-health-on-teeth/ Accessed 30 November 2025.

[26] Mishu MP Aggarwal V Shiers D Peckham E Johnston G Joury E. Developing a consensus statement to target oral health inequalities in people with severe mental illness. Health Expect. (2024) 27(4):e14163. 10.1111/hex.14163 39097761 PMC11297907

[27] Hall JP LaPierre TA Kurth NK. Oral health needs and experiences of Medicaid enrollees with serious mental illness. Am J Prev Med. (2018) 55(4):470–9. 10.1016/j.amepre.2018.05.013 30126670

[28] AlHadi AN AlAteeq DA Al-Sharif E Bawazeer HM Alanazi H AlShomrani AT. An Arabic translation, reliability, and validation of the patient health questionnaire in a Saudi sample. Ann Gen Psychiatry. (2017) 16:32. 10.1186/s12991-017-0155-1 28878812 PMC5585978

[29] Sawaya H Atoui M Hamadeh A Zeinoun P Nahas Z. Adaptation and initial validation of the patient health questionnaire - 9 (PHQ-9) and the generalized anxiety disorder - 7 questionnaire (GAD-7) in an Arabic speaking Lebanese psychiatric outpatient sample. Psychiatry Res. (2016) 239:245–52. 10.1016/j.psychres.2016.03.030 27031595

[30] Al-Jundi MA Szentpétery A John MT. An Arabic version of the oral health impact profile: translation and psychometric properties. Int Dent J. (2007) 57(2):84–92. 10.1111/j.1875-595x.2007.tb00443.x 17506467

